# Forty Cases for Operation for Appendicitis

**Published:** 1900-12

**Authors:** Charles A. Morton

**Affiliations:** Professor of Surgery, University College, Bristol; Surgeon to the Bristol General Hospital, and the Hospital for Sick Children and Women


### FORTY CASES OF OPERATION FOR APPENDICITIS.

BY

CHARLES A. MORTON, F.R.C.S.,

*Professor of Surgery, University College, Bristol; Surgeon to the Bristol General Hospital, and the Hospital for Sick Children and Women.*

In considering the question of operation in appendicitis it is necessary to estimate the mortality of the operation and that of the disease without operation. In none of my cases, in which general peritonitis was absent at the time of operation, have the patients been any the worse for the operation.

As a perusal of the table will show, I have operated in nine cases of suppurative appendicitis, in which adhesions to the parietal peritoneum were not present, and it was therefore necessary to evacuate the pus through the general peritoneal cavity, and yet there is evidence that in no case have I produced a general peritonitis. In these cases the prevention of infection of the general peritoneal cavity will depend on the care and thoroughness with which the mass of adherent coils and omentum around the pus is shut off from the general cavity by gauze or sponge packing. If this is not efficiently done the risk of operating in such cases is undoubtedly very great. I have elsewhere[1] fully described the method which I employ in these cases, and which is based on McBurney's practice.

Even in cases in which pus can be reached without opening the general peritoneal cavity, we find when we get to a certain depth in the wound much uncertainty as to what the thickened tissue beneath may be. We fear to make an opening into the bowel which we suspect is adherent to the parietal peritoneum, and we have to work with the point of a director or the finger tip until we burrow into the abscess, and in such cases it is always with a feeling of relief I see the pus flow out. I have never in such a case made an opening into the adherent bowel instead of the abscess-sac, but I never operate in these cases without the fear that I may do so. That the tissue is resonant is not evidence that it is bowel, for these fœtid abscesses often contain gas, and even when they do not, the collection is so close to the bowel there is not true dulness unless it is very large.

It is not possible to be sure by the duration of the illness that the pus can be evacuated without opening the general peritoneal cavity. I have operated on some cases as late as the eleventh day, and found no adhesions to the parietal peritoneum. We should never operate without being prepared to shut off the general cavity by gauze or sponge packing.

Apart from the presence of general purulent peritonitis, I have had four deaths after operation.

In case 24, death was due to continued suppuration many months after operation, and life might have been saved if consent had been given to an operation for removal of the appendix.

[1] *Bristol M.-Chir. J.*, 1899, xvii. 210.

In case 13, death was almost certainly due to rupture of a large abscess into the peritoneal cavity during the struggling of anæsthesia. I had advised operation in this case two days earlier. The abscess was opened without entering the general peritoneal cavity, but although the sac was very large, there was very little pus in it. As soon as he came round from the anæsthetic he complained of great abdominal pain, and dyspnœa set in, and he died in a few hours; unfortunately no *post-mortem* could be obtained. He was a publican, and had diabetes.

In case 28, the cause of death was obscure. There was no general peritonitis. The pus was found *post-mortem* to have eroded the parietal peritoneum and to have covered the kidney. The collection, no doubt, at first lay between the cæcum and ascending colon, and the parietal peritoneum of the loin. An earlier operation would almost certainly have saved the boy's life.

Case 8 was a desperately bad one. The boy had been ill some time, there was an enormous collection of offensive pus in the iliac region, which had burrowed into the inguinal canal and simulated hernia. So much serous fluid was present in the general peritoneal cavity that shifting dulness was present in the flanks, and the boy also had pneumonia at one base. He greatly improved for a week after the abscess was opened, and then developed pyæmia and died.

I am sorry to say I have never saved, by operation, any case of appendicitis complicated by purulent peritonitis. I nearly saved one, case 37. The boy had no pain, hardly any sickness, and very little distension of the abdomen, during the week he lived, after removal of the appendix and flushing and draining the peritoneal cavity. *Post-mortem* a moderate amount of peritonitis remained.

This case was operated on early, and, together with the records of cases by other surgeons, may, I think, encourage us to operate in such cases. In a late stage of general peritonitis, operation seems to me practically hopeless,—indeed, I fear in most cases it brings about the fatal termination some hours sooner than it would otherwise occur. But as such cases are absolutely hopeless without operation, probably it is worth taking the risk of a few hours less of life in extreme illness for some chance of recovery, even though a small one.

Although I have never saved one of these cases of appendicitis, complicated by general peritonitis, I have had a recovery after flushing the abdominal cavity in a case of general peritonitis from another cause,[1] and also after the same treatment in a case in which a large pelvic abscess had given way into the general peritoneal cavity, just before I opened the abdomen, but peritonitis had not started.

I have not considered cases with serous fluid in the general

[1] *Bristol M.-Chir. J.*, 1897, xv. 325.

peritoneal cavity, even when abundant, as examples of general peritonitis, as some surgeons seem to have done, in recording recoveries after operation for general peritonitis. This condition of serous effusion into the peritoneum is not very rarely associated with appendicitis and may be recovered from by simply draining the localised collection of pus. By general peritonitis, I mean that purulent fluid, as well as other morbid conditions, is present in the general peritoneal cavity.

In operating for appendicitis, with the exception of operation performed in the quiescent period in recurrent appendicitis, I have only in one case failed to find pus.

In this case (No. 31) the illness had persisted for ten days, and very marked general tympanitic distention of the abdomen was present, and the boy was crying out with pain. It was a recurrent case, and I was able to remove the appendix with very satisfactory result.

The case shows how severe and persistent the symptoms may be in a recurrent attack.

In case 30 the condition was almost the same. The boy had been ill for three weeks, and had had previous attacks. There was very little pus, and I was able to remove the appendix with a satisfactory result.

It is necessary now to estimate, as far as possible, the risks of appendicitis without operation. If the symptoms are slight and speedily pass off, obviously the risk is *nil*, though I have found that in some cases such attacks have been present before an attack of suppurative appendicitis has occurred. The more urgent risk in appendicitis is the onset of general peritonitis, the more remote, hepatic or general pyæmia, or gradual exhaustion from prolonged suppuration and hectic. Let us take the common risk first. If the case begins with severe symptoms we cannot, from the symptoms and general condition of the patient, be sure it is not one of general peritonitis from the first. We can tell in most cases, from the condition of the abdomen, but not in all. I should regard general tympanitic distention, marked general hardness of the belly-wall, and general tenderness, with the thoracic type of breathing, as signs of general peritonitis, and an indication for immediate operation, at any rate, if the patient were seen early in the illness. But I know there are cases of appendicitis in which

### TABLE OF FORTY CASES OF OPERATION FOR APPENDICITIS.*

| No. | Initials. | Age | Seen in consultation with or sent to me at Hospital by | Result. | Date. | |
|---|---|---|---|---|---|---|
| | | | *Cases in which an abscess could be drained without opening the general peritoneal cavity:* | | | |
| 1 | M. ... | 18 | General Hospital ... | Recovered in a month ... | July, 1894 | Fæcal discharge third day—lasted a week. |
| 2 | J. D. ... | 17 | General Hospital ... | Healed in 6 weeks ... ... | April, 1895 | Temperature 103° and 104° second and third day, then fell to 99°–100° for 24 hours, then increasing daily till an abscess opened on twelfth day. |
| 3 | R. P. ... | 11 | General Hospital ... | Healed in 6 weeks ... ... | July, 1895 | |
| 4 | Mr. C. | 20 | Dr. Belfield ... ... | Recovered ... ... ... | June, 1896 | Sinus did not close for several months. |
| 5 | W. G.... | 1 | General Hospital ... | Recovered ... ... ... | Oct., 1896 | Extra-peritoneal abscess extending into thigh after second attack. Discharged well two months after operation: discharge recurred, but sinus finally healed. |
| 6 | E. T. ... | 7 | General Hospital ... | Recovered ... ... ... | Sept., 1897 | Abscess very large, and reached from Poupart's ligament to well under ribs. Occasional fæcal discharge for two months. Did not close for five months. |
| 7 | Miss W. | 40 | Dr. Colman ... ... | Recovered ... ... ... | Sept., 1897 | Sinus persisted for two years; but patient got about. Two appendix concretions discharged, and sinus at once healed. |
| 8 | G. P. ... | 7 | Mr. W. F. Carter ... | Died ... ... ... ... | April, 1898 | Pneumonia present at one base at time of operation. Improved greatly for a while after abscess opened, and then pyæmia supervened. |
| 9 | Mr. C. | 35 | Dr. Mather ... ... | Recovered ... ... ... | May, 1898 | Some fæcal discharge for a few weeks. Sinus closed some weeks later. |
| 10 | A. W.... | 24 | Dr. Belfield ... ... | Recovered ... ... ... | Feb., 1899 | Sinus persisted, and appendix removed in May. Sinus did not close until many months later. Never any fæcal discharge. |
| 11 | Miss B. | 20 | Mr. A. W. Peake ... | Recovered ... ... ... | April, 1899 | Sinus persisted several months. |
| 12 | C. ... | 10 | Mr. A. W. Peake ... | Recovered ... ... ... | Aug., 1899 | Sinus persisted several months. |
| 13 | Mr. G. | 42 | Dr. Belfield ... ... | Died ... ... ... ... | Aug., 1899 | Diabetes. Very large abscess; ten days duration. Refused operation two days earlier. Died a few hours after operation, probably from rupture of abscess into peritoneum in struggling under anæsthetic. |
| 14 | A. B. ... | 21 | General Hospital ... | Recovered ... ... ... | Aug., 1899 | Secondary abscess found in pelvis, and felt per rectum; but discharged through old sinus, and sinus closed; has had but two recurrent attacks since. |
| 15 | A. G. ... | 13 | Mr. J. W. Taylor ... | Recovered ... ... ... | Mar., 1900 | Second attack. Sinus persisted some months. |
| 16 | Mr. M. | 60 | Mr. F. Peake ... ... | Recovered ... ... ... | Mar., 1900 | Pus extra-peritoneal, under cæcum. Discharged six weeks after operation, practically healed. |
| 17 | H. S. ... | 20 | Dr. McKenzie... ... | Recovered ... ... ... | April, 1900 | Sinus closed two months after operation. |
| 18 | A. H. ... | 20 | General Hospital ... | Recovered ... ... ... | April, 1900 | Discharged seven weeks after operation. Sinus not then closed. |
| 19 | A. W. B. | 32 | General Hospital ... | Recovered ... ... ... | Aug., 1900 | Many previous attacks. Illness of three weeks duration. Small quantity of extra-peritoneal pus. Discharged practically healed one month after operation. |
| 20 | Miss H. | 48 | Mr. A. W. Peake ... | Recovering ... ... ... | Oct., 1900 | |

| | | | | | | |
|---|---|---|---|---|---|---|
| | *Cases in which it was necessary to operate in general peritoneal cavity, and shut it off with gauze or sponge packing before evacuating the pus:* | | | | | |
| 21 | A. W.... | 22 | General Hospital ... | Healed in 5 weeks ... ... | May, 1895 | |
| 22 | W. T... | 8 | General Hospital ... | Recovered ... ... ... | Feb., 1896 | Only adherent to parietal peritoneum at one spot. In peeling off peritoneum here, it leaked. Pus caught on sponges. No infeetion of general cavity. Discharged one month after operation, practically healed. |
| 23 | A. W.... | 10 | Children's Hospital... | Recovered ... ... ... | May, 1897 | Pus escaped on manipulation of adherent coils and appendix after peritoneal cavity opened, and therefore general cavity flushed out. Appendix removed. Secondary abscess in liver drained. Case reported, *Bristol M.-Chir. J.*, 1897, xv. 323. |
| 24 | Mr. C. | 16 | Mr. J. W. Taylor ... | Died ... ... ... ... | Mar., 1898 | Sinus persisted, and fresh foci of suppuration formed. Parents refused to have appendix removed. Died six months after first operation. |
| 25 | W. B.... | 10 | Children's Hospital... | Died ... ... ... ... | Dec., 1898 | Died two days after operation. No general peritonitis at post-mortem. Perforated appendix. Pus in abscess. Sac lay in contact with the kidney, on which were patches of lymph. |
| 26 | Mr. A. | 30 | Dr. Albert T. Morgan.. | Recovered ... ... ... | April, 1899 | Pelvic abscess in a man, almost certainly appendicular in origin. |
| 27 | R. O. ... | 32 | Dr. Maddison ... ... | Recovered ... ... ... | June, 1899 | Not adherent to parietal peritoneum after eleven days. Fæcal discharge for 24 hours, beginning second day after operation. Discharged from hospital practically healed six weeks after operation. |
| 28 | W. D.... | 26 | General Hospital ... | Recovered ... ... ... | Oct., 1899 | Not adherent to parietal peritoneum by eleventh day. Discharged five weeks after operation, practically healed. |
| 29 | Mr. P. | 19 | Dr. Ambrose ... ... | Recovered ... ... ... | Dec., 1899 | |
| 30 | F. F. ... | 13 | Dr. Aubrey ... ... | Recovered ... ... ... | April, 1900 | Third attack of three weeks duration. Very little pus. Appendix removed. |
| | | | | *A case in which the appendix was removed, but no pus found:* | | |
| 31 | L. B. ... | 13 | Dr. Maddison... ... | Recovered ... ... ... | June, 1899 | Three previous attacks. Severe attack of ten days duration. Perforated appendix removed. Dry adhesions. |
| | | | | *Cases of recurrent appendicitis operated on after subsidence of attack:* | | |
| 32 | G. H.... | 19 | Dr. Wallace ... ... | Recovered ... ... ... | Mar., 1897 | Five attacks. |
| 33 | C. P. ... | 32 | Dr. Markham Skerritt | Recovered ... ... ... | Aug., 1897 | Six attacks. Had valvular heart disease; but took ether well. |
| 34 | T. R. ... | 33 | Mr. T. M. Carter ... | Recovered ... ... ... | Mar., 1898 | Three attacks. Movable kidney on same side operated on at later date. |
| | | | | *Cases complicated by general purulent peritonitis:* | | |
| 35 | Female | 19 | General Hospital ... | Died ... ... ... ... | June, 1894 | Flushing and drainage. |
| 36 | Male ... | 17 | General Hospital ... | Died ... ... ... ... | July, 1894 | Flushing and drainage. |
| 37 | Male ... | 8 | Children's Hospital... | Died ... ... ... ... | Feb., 1896 | Flushing and drainage. Lived a week after operation. |
| 38 | V.W.H. | 13 | Dr. Colman ... ... | Died ... ... ... ... | June, 1897 | Flushing and drainage. |
| 39 | Mr. H. | 36 | Dr. McKenzie ... | Died ... ... ... ... | Sept., 1895 | An abscess, shut off from the general cavity, was drained in Sept. 1895. Two concretions came from the sinus five weeks later, and sinus did not close for four months; then got quite well. Attack of general peritonitis six weeks later; appendix then removed. |
| 40 | A. G. ... | 42 | Dr. Cotton ... ... | Died ... ... ... ... | Aug. 1900 | Flushing and drainage. |

* These are all the cases I have operated on up to the time this paper was written.

the typical signs of peritonitis are absent, and yet general peritonitis exists from the first. I remember operating in such a case after the boy had been under careful observation for days as a case of simple appendicitis. Murphy,[1] of Chicago, McBurney,[2] and Morris,[3] of New York, point out this liability to error. They say general peritonitis cannot be excluded in a severe case with only symptoms of appendicitis. Mr. Butlin also has drawn attention to the same liability to error in diagnosis.[4]

On the other hand, if we operate early on every severe case, in which only signs of appendicitis are present, we shall, undoubtedly, operate on many which would recover without, and yet I should say—as has been said by other surgeons—the risk of operation is less than the risk of waiting: for if general peritonitis is present the chance of recovery lies in early operation. Some would no doubt rather take the risk of waiting for recovery, as the probable termination of the case would be recovery without operation. By early operation I mean operation in the first 48 hours, and I have had no experience of such. It is seldom I am asked to see the patient, unless either general peritonitis is suspected, or the case has failed to improve after some days of treatment. It seems to me, however, that if an operation is performed at this time, it would not be more difficult to isolate the coils of bowel adherent around a collection of pus within the general peritoneal cavity, than a few days later, and if no pus was found the appendix could be removed and the attack cut short and recurrence prevented.

But if the case does not materially improve by the third or fourth day, I believe the probability that pus has formed is great, and once pus has formed we have to face the risk that it may not remain shut in by adhesions, but may infect the general peritoneal cavity. I believe our rule should be to operate in all these cases—they often do recover without,—but still I think there is considerable risk in leaving them alone. There is the double risk that after all the disease may be

[1] *J. Am. M. Ass.*, 1894, xxii. 302 *et seq.*

[2] *Med. Rec.*, 1895, xlvii. 385. [3] Lectures on Appendicitis, 1895.

[4] *St. Barth. Hosp. Rep.*, 1899, xxxiv. 84.

general peritonitis from the first, or if our diagnosis is correct and the disease is only appendicitis yet pus is very likely to have formed and may become extravasated into the general peritoneal cavity. I have records of two cases in which a localised appendicular abscess ruptured and set up general peritonitis, one in which without rupture general peritonitis supervened, and four in which the symptoms and signs pointed more to simple appendicitis than general peritonitis, but the latter condition was found at the operation. On the other hand, I advised operation in two cases which recovered without. In both symptoms persisted after the fourth day, with well-marked swelling in the appendix region. Whether they have had recurrences or not I do not know. I, however, still think the safer course would be to operate in such cases. But if the case is progressing towards recovery, even when seen as late as several days after the onset, and even though the onset were severe, it is so extremely probable that the patient will recover, I do not think we could advise operation. I have seen several such cases in consultation with a view to operation, and decided not to operate. But I must say I do not feel quite safe in leaving even these cases alone. I have operated for general peritonitis on a patient who was supposed to have practically recovered from an attack of appendicitis by the medical man in charge of the case, and some years ago I made a *post-mortem* examination on a young man—a patient of another practitioner —who was supposed to be progressing very favourably, when he got much worse and very quickly died a week after the onset of his illness, and we found a pelvic abscess from perforated appendix, which had ruptured and set up general peritonitis.

If a patient had symptoms of early collapse, and yet had only abdominal signs of appendicitis, I should unhesitatingly recommend operation, as general peritonitis might very likely be present. If the symptoms were severe at the onset—intense general pain and frequent vomiting,—and both persisted for more than twenty-four hours, I know from my experience of such cases that only appendicitis may be present, but I should not feel quite sure that general peritonitis was absent, even though the abdomen was soft and only slightly distended, the temperature not much raised and the pulse under 100; and I should therefore consider the safer course to adopt would be to operate. I should be inclined to tell the patient he would very likely recover without, but I should consider it was on the whole a safer course to operate. If the symptoms had persisted for several days when I saw the case I should probably say the same thing with regard to prognosis without operation, but should express a somewhat stronger opinion with regard to the advisability of operation. It is very difficult to define what we consider the right treatment for all cases—we must consider the individual case, but I think I may say that in many severe, and in all persistently severe cases, operation is the safest course to adopt. In a case I could diagnose with certainty as one of either mechanical obstruction or early general peritonitis, I should recommend operation as practically the only chance. It is so different in even severe cases of appendicitis, for the patient may very likely recover without operation. He will almost certainly recover with skilful operation, and considerable risk will be avoided.

What is the value of the presence or absence of a localised swelling in the appendix region in deciding for or against operation? In the cases which appear to be only appendicitis, but really are cases of general peritonitis from the first, no local swelling is present. At the recent discussion on appendicitis at the Medical Society of London, a surgeon expressed the opinion that cases in which the symptoms were severe but no definite swelling was present in the cæcal region were really the most dangerous ones.[1] In cases seen after the first few days the absence of swelling, either on examination of the abdomen or examination per rectum, would I consider be evidence that there was not a collection of pus, and therefore that there was no need for operation on that ground. But it would be needful to exclude a pelvic swelling by rectal examination; and there must not be so much rigidity in the abdominal wall and so much tenderness as to make a thorough examination impossible. But a definite local swelling often disappears, and the patient gets quite well from the attack. I do not therefore think the presence of

[1] Marmaduke Sheild, *Lancet*, 1900, i. 615.

a definite swelling even of some size sufficient indication alone for operation if the patient is clearly improving in other ways. But the steady increase of such a swelling, even with a normal temperature and certainly with a high temperature, I should regard as a very strong indication of abscess formation. The recovery of a patient from an attack of appendicitis without operation is not evidence that no abscess was formed. In the records of some cases of recurrent appendicitis operated on in the quiescent period, custard-like masses are found, which can be nothing else than dried-up pus. Mr. Butlin, in his paper on appendicitis already referred to, mentions the discovery of collections of pus in these recurrent cases.[1] It is because these quiet abscesses may remain after apparent recovery from an attack of appendicitis that I fear the practice which I at present adopt, of not operating if the attack seems passing off, is not altogether a safe one.

The other risks of appendicitis—hepatic or general pyæmia—are not sufficiently frequent to weigh very much with us in deciding on operation, though they should make us realise the need for early evacuation of pus. I have successfully drained a hepatic abscess after previous removal of the appendix for perforation and suppuration around.[2]

In a few of my cases, and in those of other surgeons, a sinus persisted for some time after the abscess was opened. No doubt this continued suppuration would be avoided if we removed the appendix in all cases at the time we evacuated the pus. But this means in most cases breaking down the protective adhesions amongst the coils around the appendix and the pus, on which the safety of the general peritoneal cavity from infection depends—a risk which, as I have written elsewhere,[3] most surgeons refuse to take. Now and again it may be possible to remove the appendix without destroying these adhesions, and in such cases we ought to remove it, but they are the exception.

If suppuration persists and a sinus remains, and fresh abscesses form, so that the patient cannot get about, and perhaps suffers from hectic fever, should we take some risk and remove

[1] *Loc. cit.*, 83. [2] *Bristol M.-Chir. J.*, 1897, xv. 323.
[3] *Ibid.*, 1899, xvii. 213.

the appendix, notwithstanding the neighbouring suppuration? I think we should. I did so in one case which ended in recovery, and I wished to do so in another, but the relations would not take the risk of operation, and the young man died. In both these cases the patients remained ill from the continued suppuration. The operation was I think the most difficult I ever performed, and I despaired at one time of getting the appendix away, but at last I succeeded. In this case the sinuses were all most carefully sponged dry before the peritoneal cavity was opened, and the region of operation was packed with gauze at the close.

Even in cases in which a small sinus persists, but does not prevent the patient getting about, it may become a question in time whether the appendix should not be removed. As a rule, however, in these cases the sinus tends to close. I am sure fæcal discharge, occurring a short time after operation, from the abscess sac, and due to spontaneous perforation of the softened bowel forming the wall, need never cause serious anxiety. I have seen it in several cases, and it always soon ceases. It usually comes on a few days after the abscess has been opened.

